# Deep Learning Aided Neuroimaging and Brain Regulation

**DOI:** 10.3390/s23114993

**Published:** 2023-05-23

**Authors:** Mengze Xu, Yuanyuan Ouyang, Zhen Yuan

**Affiliations:** 1 Center for Cognition and Neuroergonomics, State Key Laboratory of Cognitive Neuroscience and Learning, Beijing Normal University, Zhuhai 519087, China; 2 Centre for Cognitive and Brain Sciences, Institute of Collaborative Innovation, University of Macau, Macau SAR 999078, China; 3 Nanomicro Sino-Europe Technology Company Limited, Zhuhai 519031, China; 4 Jiangfeng China-Portugal Technology Co., Ltd., Macau SAR 999078, China

**Keywords:** artificial intelligence, deep learning, medical imaging, neuroimaging, brain regulation

## Abstract

Currently, deep learning aided medical imaging is becoming the hot spot of AI frontier application and the future development trend of precision neuroscience. This review aimed to render comprehensive and informative insights into the recent progress of deep learning and its applications in medical imaging for brain monitoring and regulation. The article starts by providing an overview of the current methods for brain imaging, highlighting their limitations and introducing the potential benefits of using deep learning techniques to overcome these limitations. Then, we further delve into the details of deep learning, explaining the basic concepts and providing examples of how it can be used in medical imaging. One of the key strengths is its thorough discussion of the different types of deep learning models that can be used in medical imaging including convolutional neural networks (CNNs), recurrent neural networks (RNNs), and generative adversarial network (GAN) assisted magnetic resonance imaging (MRI), positron emission tomography (PET)/computed tomography (CT), electroencephalography (EEG)/magnetoencephalography (MEG), optical imaging, and other imaging modalities. Overall, our review on deep learning aided medical imaging for brain monitoring and regulation provides a referrable glance for the intersection of deep learning aided neuroimaging and brain regulation.

## 1. Introduction

In recent years, the medical field has become one of the most important research and application fields in the artificial intelligence (AI) industry. Machine learning and its subset, deep learning, are branches of AI, and have shown promising findings in the medical field, especially when applied to imaging data, which have been used in radiological diagnosis, bioinformatics, genome sequencing, drug development, and histopathological image analysis [1,2]. Segmentation of brain disease lesions can provide imaging biomarkers of disease burden that can help monitor disease progression and the imaging response to treatment [3]. Particularly for histopathological diagnosis, AI has exhibited a potential ability that matches that of medical experts. The AI aided neuroimaging and brain regulation generally employed distinguished deep learning and conventional machine learning (include rule-based learning). Classical machine learning methods such as support vector machine (SVM) or random forest require a well-prepared feature engineering procedure, in other words, need to manually segment morphological features and select import features, which is extremely time consuming and tends to show a large performance difference between different operators [4]. As AI techniques continue to be refined and improved, deep learning has been proposed to dramatically change the health care monitoring and regulation of the brain [5], which can not only improve the reconstruction accuracy of neuroimaging and achieve fast imaging, but also mine a large amount of pathological and genetic data by processing and cross-referencing health and medical big data such as images, pathology, and genes, and help pathologists to evaluate pathological sections faster to improve the efficiency and prognosis of disease diagnosis [6]. Deep learning is a special type of machine learning. The advantage of deep learning is to utilize a neural network-like engineering architecture that can detect and extract import features automatically, whose predicting label and assessment algorithm has developed several varieties including prognosis, immune-score, microsatellite instability, histological subtyping, microenvironment analysis and segmentation, etc. Extensive studies have revealed high accuracies and provided excellent examples of deep learning’s potential in brain health care [7,8,9]. Thanks to advancements in neuroimaging technology, numerous neuroimaging datasets of brains have been gathered from multiple imaging facilities utilizing various scanners and imaging protocols. These datasets have been collected to study both typical and atypical brain development [10]. Recent advancements in medical imaging of the brain have greatly solved challenges from biopsy—an invasive, unrepeatable technique that usually ignore heterogeneity within parenchyma. It uses data characterization algorithms to convert conventional imaging information into data matrices by modern linear algebra and statistics, which can be further extracted into information revealing a certain malady.

Currently, deep learning-aided medical imaging is becoming the hot spot of AI frontier application and the future development trend of precision neuroscience. Clinical decision-making, an essential element of medicine, involves judgement with the integration of comprehensive data [11]. Especially in brain or central nervous system diseases, there are unique challenges in medical decision-making due to their diverse forms and progression as well as the need to consider individual patient factors such as their ability to receive treatment and response to it. The early detection of cancer is crucial in saving thousands of lives. Targeted therapy for cancer heavily relies on its grading [12]. Due to the invasive, time-consuming, and expensive nature of cancer diagnosis, there is an urgent need to develop non-invasive, cost-effective, and efficient tools to characterize and estimate the grade of brain cancer. MRI, PET/CT, EEG/MEG, optical imaging, and other imaging modalities offer quick and safer options for tumor detection during brain scans. The utilization of deep learning in molecular diagnosis, prognosis, and treatment monitoring has resulted in the creation of a structured resource for radiogenomic analysis of brain or central nervous system diseases. Besides greatly reducing the scan time of neuroimaging methods like MRI and PET/CT [13], the deep learning aided medical images acquired better signal to noise ratio, higher contrast-to-noise ratio, and stronger brain or central nervous system disease lesion detection ability. Therefore, radiomics was thought to be the bridge between medical imaging and personalized medicine [14], which is a quantitative approach to medical imaging that involves the extraction and analysis of large amounts of quantitative data from medical images such as MRI and PET/CT. These data are then used to create predictive models that can help doctors make more accurate diagnoses, predict treatment outcomes, and develop personalized treatment plans. Deep learning is a subset of machine learning that uses artificial neural networks to analyze and learn from data. In radiomics, deep learning algorithms can be trained on large datasets of medical images to identify patterns and features that are not visible to the human eye. This can help radiologists and other medical professionals to more accurately diagnose and treat a wide range of medical conditions including cancer, neurological disorders, and cardiovascular disease. By applying deep learning to medical imaging, radiomics has the potential to revolutionize the way we diagnose and treat disease. It can help to identify early-stage disease, predict which patients will respond best to which treatments, and develop new, personalized treatment plans based on a patient’s unique genetic and environmental factors.

Deep learning algorithms have been used to accurately segment brain tumors in MRI scans, diagnosis of Alzheimer’s disease, brain–computer interfaces (BCI), brain stimulation, and so on. For example, a study used a deep learning algorithm to segment brain tumors in MRI scans with high accuracy. The algorithm was trained on a large dataset of MRI scans and was able to accurately segment tumors, even in cases where the tumor was irregularly shaped or located close to the brain’s surface [15]. In another study, Xiaojun Bi and Haibo Wang proposed a discriminative version of a contractive slab and spike convolutional deep Boltzmann machine model (DCssCDBM) with a multi-task learning framework via EEG spectral images based on identification and verification tasks for overfitting reduction for the first time, and the method accurately predicted whether a patient had Alzheimer’s disease or not [16]. Deep learning algorithms have also been used to improve the accuracy and speed of BCI systems, which allow people to control computers or other devices with their thoughts. For example, a study used a novel deep learning algorithm to improve the accuracy of a BCI system for hand movement detection and the algorithm was able to detect hand movements with high accuracy [17]. Deep learning algorithms have also been applied to optimize brain stimulation techniques such as transcranial magnetic stimulation (TMS), which is used to treat depression and other mental health conditions. For example, a study used connectivity measures and an ensemble of pre-trained deep learning models to predict the treatment outcome of repetitive TMS in major depressive disorder to improve the treatment efficacy and reduce health care costs. The methodology possesses effective connectivity, used for transforming EEG signals to images, and provides an informative feature map [18].

**Figure 1:**
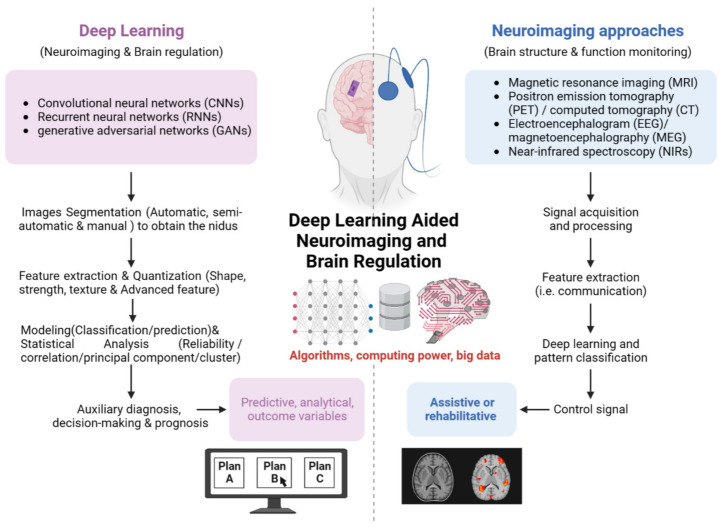
Schematic illustration of the review on deep learning aided neuroimaging and brain regulation.

Deep learning holds the potential to greatly improve the brain medical image quality, metastasis detection, radiogenomics, and treatment response monitoring, which can assist with volumetric delineation of brain lesions over time, extrapolation of the genotype and biological course from radiographic phenotype, prediction of clinical outcomes, and assessment of the impact of disease and treatment on the surrounding encephalic region [19,20]. By automating the initial image interpretation, deep learning may revolutionize the clinical workflow of radiographic detection, management decisions, interventions, and follow-up care in ways yet to be envisioned [21]. With more and more deep learning aided medical imaging and therapy decision-making strategies entered into the national authoritative professional society and cancer diagnosis and treatment guidelines that are followed by across the country, deep learning assisted theranostic methods are widely recognized by clinical doctors and lay a solid foundation for its large-scale clinical application. Therefore, in vivo image acquisition and data signal analysis, data grouping storage, separate and detailed recording of each sample’s experimental data, and through the professional analysis module for high-precision quantitative analysis of imaging data, can analyze multiple groups of data at the same time to ensure the consistency of the experimental data [22]. Although there are challenges to overcome such as inter-scanner variability, the need for benchmark datasets, and prospective validations for clinical applicability, there is a significant opportunity for the development of optimal solutions for brain or central nervous system disease stratification. These solutions can provide immediate recommendations for further diagnostic decisions, the guidance of deep brain stimulation target identification and personalized treatment plan optimization [23,24,25]. In a word, using deep learning to assist medical imaging for brain theranostics has the characteristics of objectivity, high-accuracy, and high efficiency beyond the abilities of human judgement from qualitative to quantitative imaging. This review is schematically illustrated in Figure 1.

## 2. Evolution and Classification of Deep Learning Assisted Medical Imaging

### 2.1. Evolution of Artificial Intelligence in Medical Imaging

The development of AI and deep learning can be traced back to the 1950s and 1960s [26,27]. Early AI research included rule-based systems that relied on human-written rules to solve specific problems. However, the capabilities of these systems were very limited because they needed to manually write all the rules, and they struggled to cope with complex and uncertain situations [28]. In the 1980s and 1990s, machine learning became popular as improved computer power and large amounts of data became available. The idea is for computers to learn patterns from data so they can better handle new data. One of the important machine learning techniques is neural networks, which are algorithms inspired by the human nervous system. However, early neural networks were very shallow, with only a few layers, limiting their capabilities. With the development of deep learning algorithms, neural networks have become deeper and more complex, which can better handle large amounts of data and have achieved many breakthrough results. The most representative examples are the application of deep learning in image and speech recognition as well as its success in fields such as natural language processing and machine translation. In recent years, with the improvement in computer performance and algorithms, the application scope of AI and deep learning has been expanding, gradually penetrating into various fields and achieving more and more success [29], showing its advantage in real scenarios including lung nodules in chest CT, neuroimaging, mammography, and so on.

AI is transforming medical imaging and driving it forward toward the future at a rapid pace. The evolution of AI in medical imaging has been a game-changer in the field of health care. Medical imaging is a critical component of medical diagnosis and treatment, and AI has significantly improved the accuracy, efficiency, and speed of medical imaging processes [30,31]. The early application of AI in medical imaging focused on computer-aided diagnosis, which involved using algorithms to detect and classify lesions or abnormalities in medical images [32,33,34]. However, with the rise of deep learning, the application of AI in medical imaging has become more sophisticated, enabling the development of predictive models, automatic image segmentation, and even image synthesis [35]. One of the significant advantages of AI in medical imaging is its ability to analyze vast amounts of data quickly and accurately, especially in image segmentation, registration, detection, and recognition. This has led to the development of systems that can detect and diagnose diseases with high accuracy such as lung cancer, head and neck cancer, breast cancer, and diabetic retinopathy [36]. Moreover, AI has facilitated the automation of repetitive tasks, freeing up time for medical professionals to focus on more complex cases [37,38]. AI has also enabled the development of personalized medicine, where treatments can be tailored to individual patients based on their genetic makeup, medical history, and imaging data. This has led to better patient outcomes, reduced costs, and improved overall health care efficiency. However, the adoption of AI in medical imaging is not without its challenges. One of the main concerns is the potential for bias in AI algorithms, which can lead to inaccurate diagnoses or treatment recommendations [1]. Moreover, there is a need for transparent and ethical AI practices including the development of regulatory frameworks, to ensure that AI is used safely and effectively. Generally, the evolution of AI in medical imaging has revolutionized the field of health care, with the potential for improving patient outcomes, reducing costs, and increasing overall efficiency. The continued development and adoption of AI in medical imaging will undoubtedly lead to further advancements in health care and personalized medicine, which has the potential to not only revolutionize traditional medical imaging, but also enhance clinical workflows and transform various aspects of the health care industry.

### 2.2. Convolutional Neural Networks (CNNs)

The application of convolutional neural networks (CNNs) in medical imaging, especially in brain monitoring and modulation, has made remarkable progress [39,40,41]. CNNs are deep learning algorithms, which has been widely used in image recognition and classification. It can automatically extract features and patterns from a large amount of data. In terms of brain monitoring, CNNs can be used to analyze neuroimaging data such as MRI and CT scan results as well as physiological data such as EEG and MEG. CNNs can automatically identify brain structures and activity patterns to help doctors diagnose and treat them [42,43,44,45]. In terms of brain regulation, CNNs can be used in brain–computer interface technology, which is a technology that converts electrical brain signals into machine commands [46]. Using CNNs to analyze EEG signals, EEG patterns such as event-related potentials and bands can be identified to help people realize brain-controlled devices and applications such as wheelchairs and games. In addition, CNNs can also be used to predict and monitor the progression of brain diseases and response to treatment. By analyzing brain images and physiological data, CNNs can automatically identify pathological patterns and features to predict disease progression and treatment effects [47,48,49]. In general, the application of CNNs in medical imaging, especially in brain monitoring and regulation, provides doctors and patients with more accurate, faster, and more effective treatment methods, which is expected to bring more progress and innovation to the medical health field in the future.

### 2.3. Recurrent Neural Networks (RNNs)

The application of recurrent neural networks (RNNs) in medical imaging, especially in brain monitoring and regulation, has also made some progress [50,51,52]. Unlike CNN, cyclic neural networks can process data with a sequential structure such as time series and speech signals [53,54,55]. In terms of brain monitoring, RNNS can be used to analyze time series data such as EEG and MEG as well as functional magnetic resonance imaging (fMRI) data. RNNs can recognize brain wave patterns and trends, and make predictions and classifications based on the dynamic nature of the data. In terms of brain regulation, RNNS can be used in brain–computer interface technology (BCIs), which is a technique that converts electrical signals in the brain into machine instructions. By analyzing EEG signals with a RNN, patterns and dynamic changes of EEG can be identified to help people realize more refined and flexible brain control devices and applications. In addition, RNNS can be used to predict brain diseases and monitor therapeutic responses [56,57,58]. By analyzing time series and dynamic data, RNNs can automatically identify brain pathological patterns and features to predict disease progression and treatment effects [59]. In general, the application of RNN in medical imaging, especially in brain monitoring and regulation, provides doctors and patients with a more comprehensive, precise, and dynamic treatment style, which is expected to bring more progress and innovation to the medical health field in the future.

### 2.4. Generative Adversarial Networks (GANs)

**Figure 2:**
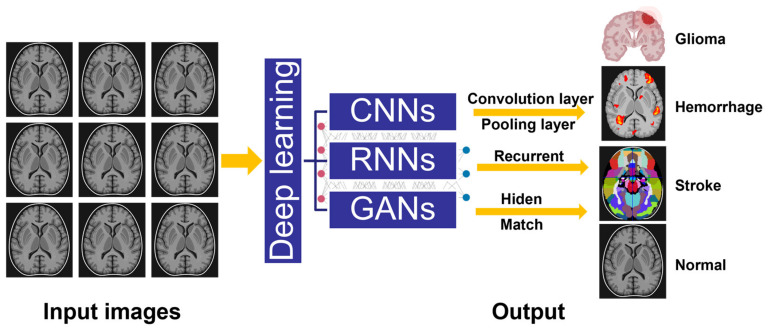
Applications of different neuron network-assisted brain health care monitoring.

The application of a generative adversarial network (GAN) in medical imaging, especially in brain monitoring and regulation, has also received more and more attention and research. GAN is a deep learning algorithm that can be used to generate images and data with specific characteristics and attributes [60,61,62,63,64]. In terms of brain monitoring, GAN can be used to generate virtual brain images with specific brain structures and activity patterns. This can help doctors understand the relationship between different brain structures and activity patterns, and the impact on different brain diseases [65,66,67,68,69,70]. In addition, GAN can also be used to synthesize brain images with different pathological features and trends to help doctors diagnose and treat diseases. In terms of brain regulation, GAN can be used in brain–computer interface technology (BCI) to help train and optimize brain-control devices and applications by generating virtual images and data with specific patterns of brain activity. In addition, GAN can be used to generate virtual images and data with specific brain pathological patterns to help researchers explore mechanisms and treatments for brain diseases [71,72,73,74]. Broadly speaking, the application of GAN in medical imaging, especially in brain monitoring and regulation, provides a more comprehensive, targeted, and innovative treatment for doctors and patients, and is expected to bring more progress and innovation to the medical health field in the future. The applications of different neuron network-assisted brain health care monitoring are illustrated in Figure 2.

## 3. Deep Learning Aided Neuroimaging for Brain Monitoring and Regulation

Deep learning has shown tremendous potential in the field of neuroimaging and brain regulation. Neuroimaging techniques such as MRI, CT, PET/CT, EEG/MEG, optical imaging, and other imaging modalities generate large amounts of comprehensive and complex data, which can be challenging to analyze and interpret. Deep learning techniques such as CNNs, RNNs, and GANs have been proven to be effective in extracting meaningful information from these data and transforming the neuroimaging from qualitative to quantitative imaging modality. The aforementioned information is merged with additional patient data and processed using advanced bioinformatics software to create models that could potentially enhance the accuracy in the diagnosis, prognosis, and prediction for brain monitoring and regulation.

### 3.1. Deep Learning Assisted MRI

MRI scans of the brain are considered the most effective approach for identifying chronic neurological disorders such as brain tumors, dementia, stroke, and multiple sclerosis. They are also the preferred method for detecting conditions affecting the pituitary gland, brain vessels, inner ear organs, and eyes due to their high sensitivity. In recent years, several deep learning-based medical image analysis methods have been introduced to facilitate health monitoring and diagnosis using brain MRI scans [75,76,77]. One of the primary applications of deep learning in neuroimaging by MRI is the identification and classification of neurological disorders. For example, CNNs have been used to accurately diagnose Alzheimer’s disease, Parkinson’s disease, and multiple sclerosis by analyzing MRI scans. Deep learning has also shown potential in identifying different stages of brain development, detecting early signs of neurological disorders, and predicting the progression of these disorders.

Recent advancements in the classification of gliomas based on biological genotypes, coupled with the utilization of computational deep learning models based on multi-modal MRI biomarkers, offer a promising avenue for customized and effective treatment plans. In this regard, deep learning-based assessment of gliomas using hand-crafted or auto-extracted features derived from MRI has emerged as a critical tool, as genomic alterations can be correlated with MRI-based phenotypes [78]. Deep learning algorithms have been extensively explored for the purpose of classifying neurodegenerative diseases using medical imaging techniques such as magnetic resonance imaging. Utilizing CNNs on MRI data has emerged as a promising technique for achieving exceptional levels of precision in predicting the progression of neurological conditions such as brain tumors, Alzheimer’s disease, multiple sclerosis, and stroke by capturing image features that are not detectable using traditional methods. However, little attention has been given to utilizing post-mortem immunofluorescence imaging studies of patients’ brains for this purpose. These studies have the potential to be a valuable tool in detecting abnormal chemical changes or pathological post-translational modifications of the Tau polypeptide. Therefore, L. Diaz-Gomez et al. proposed a CNN pipeline that utilized transfer learning to analyze post-mortem immunofluorescence images with different Tau biomarkers for the classification of Tau pathology in Alzheimer’s disease and progressive supranuclear palsy. The ResNet-IFT architecture was used to generate models, and interpretability algorithms such as Guided Grad-CAM and occlusion analysis were employed to interpret the outputs of these models. They tested four different architectures to determine the best classifier, and the results showed that their design was able to classify diseases with an average accuracy of 98.41%. Additionally, they were able to provide an interpretation of the classification, which included different structural patterns in the immunoreactivity of the Tau protein in NFTs present in the brains of patients with progressive supranuclear palsy and Alzheimer’s disease [79]. O. Ozkaraca et al. created a new modular deep learning model to enhance the classification accuracy of MRI images while simultaneously addressing the drawbacks of prevalent transfer learning approaches like DenseNet, VGG16, and basic CNN architectures. They employed brain tumor images from the Kaggle database to train and test their model using two distinct data splitting methods: 80% for training and 20% for testing, and 10-fold cross-validation. Although the proposed deep learning model demonstrated better classification performance compared to other transfer learning methods, it required more processing time [75]. In another study, T. Chattopadhyay et al. utilized 3D CNN to forecast Abeta+ based on 3D brain MRI data derived from 762 elderly participants (mean age: 75.1 years ± 7.6SD; 394F/368M; 459 healthy controls, 67 with MCI, and 236 with dementia) who were scanned as part of the Alzheimer’s Disease Neuroimaging Initiative. The 3D CNN accurately projected Abeta+ with a 76% balanced accuracy from T1w scans [80]. Exploring CNN-generated attention maps, which identify the most significant anatomical features used for CNN-driven decisions, holds the potential to unveil crucial disease mechanisms that contribute to the accumulation of disability. L. Coll et al. predicted a class of multiple sclerosis disability using whole-brain MRI scans as input by a 3D-CNN model, which achieved a mean accuracy of 79% and proved to be superior to the equivalent logistic regression model (77%). The model was also successfully validated in the independent external cohort without any re-training (accuracy = 71%) [81]. Perinatal arterial ischemic stroke has been linked to unfavorable neurological outcomes. However, the evaluation of ischemic lesions and the subsequent development of the brain in newborns requires time-consuming manual inspection of brain tissues and ischemic lesions. Therefore, R. Zoetmulder et al. proposed an automatic method that used CNNs to segment brain tissues and ischemic lesions in the MRI scans of infants suffering from perinatal arterial ischemic stroke. This method eliminates the need for the labor-intensive manual assessment of brain tissues and ischemic lesions [82]. This study indicates that the automatic segmentation of brain tissue and ischemic lesions in the MRI scans of patients is feasible and may allow for the evaluation of the brain development and efficacy of treatment in large datasets.

### 3.2. Deep Learning Assisted PET/CT

PET/CT provides powerful diagnosis methods for neurodegenerative disorder by identifying disease-specific pathologies. Deep learning techniques have shown great promise in enhancing PET and CT imaging for neuroimaging and brain monitoring/regulation. These techniques can help improve the accuracy, speed, and efficiency of image processing, enabling more effective analysis and interpretation of neuroimaging data. Three-dimensional CNN can be trained to denoise the PET images for each disease cohort of neurodegenerative disorders [83] and predict the diagnosis of dementia with Lewy bodies, Alzheimer’s disease, and mild cognitive impairment [84] as well as amyloid standardized uptake value ratio through PET for Alzheimer’s prognosis [85]. One example of deep learning-assisted neuroimaging is the use of convolutional neural networks (CNNs) to improve the accuracy of PET image segmentation. In one study, the researchers developed a CNN-based segmentation method that achieved higher accuracy (96%), sensitivity (96%), and specificity (94%) than the traditional methods in the evaluation of neuro images for the diagnosis of Alzheimer’s disease, which was evaluated using the ^18^FDG-PET images of 855 patients including 635 normal control and 220 Alzheimer’s disease patients from the ADNI database, thus capable of discriminating the normal control from the Alzheimer’s disease patients [86].

Another example is the use of deep learning techniques to enhance the quality and resolution of CT imaging in neuroimaging. In a study published in the journal *Radiology*, the researchers constructed and trained a deep learning-based stenosis and plaque classification algorithm for head and neck CT angiography that achieved 85.6% consistency between radiologists and the DL-assisted algorithm, and reduced the time needed for diagnosis and report writing by the radiologists from 28.8 min ± 5.6 to 12.4 min ± 2.0 (*p* < 0.001) [87]. In addition to image processing, deep learning techniques have also been used to analyze neuroimaging data such as semantic segmentation and quantification of intracerebral hemorrhage (ICH), perihematomal edema, and intraventricular hemorrhage on non-contrast CT scans of patients with spontaneous ICH [88]. Overall, deep learning techniques hold great promise for improving the accuracy, speed, and efficiency of PET and CT imaging for neuroimaging and brain monitoring/regulation. With further development and refinement, these techniques could revolutionize the field of neuroimaging and contribute to a better understanding of brain function and dysfunction.

### 3.3. Deep Learning Assisted EEG/MEG

Deep learning has become an increasingly popular tool in EEG/MEG neuroimaging and brain monitoring/regulation. With the ability to analyze large datasets and detect subtle patterns in neural activity, deep learning has shown great potential in enhancing our understanding of brain function and informing clinical applications [89,90]. Deep learning has also been used to improve brain regulation techniques such as EEG neurofeedback. EEG neurofeedback is a non-invasive technique that aims to regulate brain activity by providing real-time feedback to the patient. Deep learning algorithms can analyze EEG data in real-time, detect patterns, and provide targeted feedback to patients to help them regulate their brain activity. One of the main applications of deep learning in EEG/MEG is in the classification of brain states or disorders. For example, Chambon et al. used a CNN to classify EEG data into three different cognitive states, achieving an accuracy of up to 90% [91]. In another study, Lawhern et al. used a deep learning model to classify EEG data into different types of epileptic seizures with high accuracy, demonstrating the potential of deep learning in aiding clinical diagnosis [92].

Moreover, deep learning techniques have been used in the development of brain–computer interfaces (BCIs) that allow patients to control external devices such as prosthetic limbs or computer interfaces using their brain activity. Deep learning algorithms can extract meaningful information from EEG or fMRI data and translate it into commands for external devices. Deep learning has also been used for brain activity prediction and regulation. For instance, M. Dinov et al. used deep reinforcement learning in closed-loop behavioral-and neuro-feedback to track and optimize human performance [93], in which a deep learning model was established to predict individualized EEG signals and applied to a closed-loop system for real-time neurofeedback, achieving the successful regulation of brain activity. Recent technological advances such as wireless recording, deep learning analysis, and real-time temporal resolution have increased interest in EEG-based brain–computer interface approaches [94,95]. A deep learning model was developed for real-time decoding of MEG signals, which was applied to a brain–computer interface system for regulating motor imagery tasks [96]. Epilepsy is a chronic brain disorder in which functional changes may precede structural ones and which may be detectable using existing modalities [97]. Functional connectivity analysis using EEG and resting state-functional magnetic resonance imaging (rs-fMRI) can localize epilepsy [98,99]. Finally, deep learning has been used for feature extraction and representation learning in EEG/MEG data. For example, R. Hussein et al. used a deep learning model to learn features from raw EEG data, which were then used for the classification of epileptic seizures [100]. Similarly, Roy et al. used a deep learning smart health monitoring model with the spectral analysis of scalp EEG to automatically predict epileptic seizures [101].

In summary, deep learning has shown great potential in enhancing EEG/MEG neuroimaging and brain monitoring/regulation. By enabling accurate classification of brain states and disorders, predicting and regulating brain activity, and learning meaningful representations from EEG/MEG data, deep learning has the potential to revolutionize our understanding of brain function and inform clinical applications.

### 3.4. Deep Learning Assisted Optical Neuroimaging and Others

Optical imaging is a non-invasive technique that uses light to visualize tissue structure and function. Optical neuroimaging holds great promise for imaging guided brain regulation. For example, a study published in *Nature Neuroscience* in 2020 used deep learning to predict behavior from functional imaging data in mice, demonstrating the potential for using deep learning in real-time behavioral prediction and manipulation [102]. The non-invasive guidance of therapeutic strategies would enable the removal of cancerous tissue while avoiding side effects and systemic toxicity, preventing damage to healthy tissues and decreasing the risk of postoperative problems such as bioluminescence imaging (BLI), fluorescence imaging (FI), Cerenkov luminescence imaging (CLI), and photoacoustic imaging (PAI). BLI is always used in small animal imaging for the in vivo tracking of therapeutic gene expression and cell-based therapy. In contrast, FI is highly promising for clinical translation. The applications of FI include image-guided surgery, radiotherapy, gene therapy, drug delivery, and sentinel lymph node fluorescence mapping. CLI is a novel radioactive optical hybrid imaging strategy for animal and clinical translation. Deep learning has shown significant promise in optical imaging modalities for neuroimaging and brain regulation including photoacoustic imaging and photoacoustic tomography. Photoacoustic imaging is a hybrid imaging technique that combines the advantages of optical and ultrasound imaging. It uses laser light to generate acoustic signals, which are then used to create high-resolution images of tissue structure and function. Deep learning has been applied to photoacoustic imaging for neuroimaging, and there have been several relevant studies in this area. Using deep learning to reconstruct high-resolution images of the cerebral vasculature from photoacoustic tomography data can significantly improve image quality and reduce imaging artifacts in photoacoustic tomography [103]. In 2020, a new deep learning method called Pixel-DL was proposed by S. Guan et al., which involved pixel-wise interpolation based on the physics of photoacoustic wave propagation followed by the use of CNN to reconstruct images. Synthetic data and data from phantoms of the mouse-brain, lung, and fundus vasculature were used to train and test the model. The results showed that Pixel-DL performed similarly or better than the iterative methods and consistently outperformed other CNN-based approaches in correcting artifacts. Furthermore, Pixel-DL is a computationally efficient approach that enables real-time photoacoustic tomography rendering and improves the quality of image reconstruction for limited-view and sparse data [104].

Recent advancements in deep learning assisted optical neuroimaging have greatly solved challenges from biopsy—an invasive, unrepeatable technique that usually ignores heterogeneity within the brain. From a resolution perspective, current optical images for brain disease diagnosis include two branches of cytological and histopathological. The former cytological examination is generally inexpensive, minimally invasive, and easily repeatable compared to histopathological examination. However cytological examinations are also labor intensive and insensitive compared to histopathological examination. Image interpretation is a highly subjective task and deep learning has revealed its ability for a more objective and straightforward diagnosis. Specifically, most deep learning research has focused on optical images on a histopathological scale [105]. Deep learning began to gain more attention in the health care sector due to its promising results in recent years, which use data characterization algorithms to convert conventional imaging information into data matrices by modern linear algebra and statistics that can further be extracted into information revealing certain patterns. These deep learning approaches have been used in radiological diagnosis, bioinformatics, genome sequencing, drug development, and histopathological image analysis. Particularly for histopathological diagnosis, deep learning has surpassed that of clinical experts. The core advantage of deep learning is that it utilizes a complex neural network-like engineering architecture that can detect and extract import features automatically. The predicting label and assessment of the deep learning algorithm has developed several varieties including prognosis, PD-L1 status, microsatellite instability, histological subtyping, microenvironment analysis, and segmentation. Moreover, deep learning can solve the problem that some neuroimaging is difficult to quantify in three dimensions. However, the use of deep learning in neuroimaging and brain regulation also presents challenges. The interpretation of deep learning models is often opaque, making it difficult to understand the reasoning behind the model’s decisions. Moreover, deep learning algorithms require large amounts of data to be trained effectively, which can be challenging to acquire in the field of neuroimaging. In conclusion, deep learning has shown significant promise in various imaging modalities for neuroimaging and brain regulation including fluorescence imaging, photoacoustic imaging, and photoacoustic tomography. These studies demonstrate the potential for using deep learning to improve the image quality, reduce imaging artifacts, and develop predictive models for the diagnosis and treatment of neurological disorders.

## 4. Conclusions

In recent years, deep learning and sensing technologies have made impressive advances in medical health care monitoring. The following are several specific directions: ① Deep learning-based medical imaging diagnosis: Deep learning technology can make an intelligent diagnosis of medical images to improve the accuracy of the diagnosis. For example, deep learning algorithms can identify image features of diseases such as brain tumors and neurodegenerative disorders and provide accurate diagnosis results. ② Medical monitoring based on sensor technology: Sensor technology enables real-time monitoring of biological signals such as electroencephalography (EEG), electrocardiogram (ECG), etc. By analyzing these biological signals through deep learning technology, accurate physiological parameter measurement and abnormal detection can be achieved, providing important reference information for doctors. ③ Health management based on deep learning: Deep learning technology can analyze a large amount of health data such as biological signals, movement tracks, eating habits, etc. By analyzing these data, more accurate health management suggestions and personalized health intervention programs can be provided to the users. ④ Health risk prediction based on sensor technology and deep learning: Sensor technology can collect a large amount of physiological data such as blood pressure, blood sugar, oxyhemoglobin saturation, blood flow velocity, etc. The analysis of these data through deep learning technology can build health risk prediction models and provide users with personalized prevention and intervention recommendations. In general, the application of deep learning and sensing technology in the field of medical monitoring provides doctors and patients with more intelligent and precise services and treatments, which is expected to bring more progress and innovation in the field of medical health in the future. However, the use of deep learning in neuroimaging and brain regulation also presents challenges. The interpretation of deep learning models is often opaque, making it difficult to understand the reasoning behind the model’s decisions. Moreover, deep learning algorithms require large amounts of data to be trained effectively, which can be challenging to acquire in the field of neuroimaging. In conclusion, deep learning has shown great promise in the field of neuroimaging and brain regulation, with the potential to improve the accuracy and speed of diagnosis and the treatment of neurological disorders as well as enable new forms of brain–computer interfaces. However, the challenges associated with deep learning must be addressed to ensure that these techniques can be used safely and effectively in clinical settings. Overall, this article reviewed the recent progress of how deep learning is being applied in the medical field of neuroimaging and brain regulation. As research in this field continues to grow, we can expect to witness, and even participate in, more innovative applications of deep learning that will improve our understanding of the brain and advance our ability to treat neurological disorders.
